# Post-dural puncture headache combined with pneumocephalus secondary to vaginal delivery following epidural anesthesia: a case report

**DOI:** 10.1186/s12884-023-05861-6

**Published:** 2023-07-31

**Authors:** Xiaoyu Wu, Xiangqi Cao, Mengyuan Zhang, Qingfan Wang, Jiaxin Han, Xinyue Sun, Kang Huo, Suhang Shang, Guogang Luo

**Affiliations:** 1 First Affiliated Hospital of Xi’an Jiaotong University, No. 277 Yanta West Road, Xi’an, 710061 P.R. China; 2 Tang Du Hospital, The Fourth Military Medical University, No.1 Xinsi Road, Xi’an, 710038 P.R. China; 3 Second Affiliated Hospital of Xi’an Jiaotong University, No. 157 Xiwu Road, Xi’an, 710004 P.R. China

**Keywords:** Pneumocephalus, Post-dural puncture headache, Headache, Analgesia, Epidural

## Abstract

**Background:**

Pneumocephalus is rare in vaginal deliveries. Pneumocephalus may be asymptomatic or present with signs of increased intracranial pressure. However, parturients who received epidural anesthesia with air in their brains may experience low intracranial pressure headaches after giving birth, causing the diagnosis of pneumocephalus to be delayed. We report a case of a parturient who developed post-dural puncture headache combined with pneumocephalus secondary to vaginal delivery following epidural anesthesia.

**Case presentation:**

A 24-year-old G1P0 Chinese woman at 38 weeks gestation was in labor and received epidural anesthesia using the loss of resistance to air technique and had a negative prior medical history.

She presented with postural headache, neck stiffness and auditory changes 2 h after vaginal delivery. The head non-contrast computed tomography revealed distributed gas density shadows in the brain, indicating pneumocephalus. Her headache was relieved by bed rest, rehydration, analgesia, and oxygen therapy and completely disappeared after 2 weeks of postpartum bed rest.

**Conclusions:**

This is the first report that positional headaches after epidural anesthesia may not indicate low intracranial pressure alone; it may combine with pneumocephalus, particularly when using the loss of resistance to air technique. At this moment, head computed tomography is essential to discover other conditions like pneumocephalus.

## Background

Pneumocephalus is defined as the presence of air in the epidural, subdural, or subarachnoid space, within the brain parenchyma or ventricular cavities [1], which is most commonly seen following head and facial trauma and neurosurgery or otorhinolaryngological procedures. With the global popularity of epidural labor analgesia techniques, post-dural puncture headache (PDPH) has become a common cause of headache in parturients with an incidence ranging from 0.5% to 1.7% [2], while pneumocephalus is a rare complication of epidural anesthesia. However, the true incidence of pneumocephalus in obstetrics is very difficult to establish, mainly due to the fact that available literature largely reflects a few cases reported annually [3]. In past reports, pneumocephalus-related headache is characteristically caused by increased intracranial pressure (ICP) immediately after a dural puncture and injection of air linked to attempted epidural anesthesia utilizing the loss of resistance to air (LORA) approach [4].

However, parturients who received epidural anesthesia with a little amount of air in their brains may experience low intracranial pressure headaches after giving birth, causing the diagnosis of pneumocephalus to be delayed. Wrong diagnosis and treatment could have fatal results, such as seizures and neurological deficits. This is the first reported parturient who developed PDPH combined with pneumocephalus secondary to vaginal delivery following epidural anesthesia.

## Case presentation

A 24-year-old, 151 cm, 50 kg, gravida 1, para 0, previously healthy Chinese woman presented to the obstetrics department at 38 weeks of gestation after experiencing 2 h of frequent uterine contractions. Two days later, she was in labor and requested labor analgesia. She had no drug allergies and had never received anesthesia before. Her pre-anesthesia blood pressure was 138/67 mmHg, her heart rate was 72 beats/minute, and her respiratory rate was 21 breaths/minute. The fetal heart rate was 136 beats per minute and reactive. The course of her pregnancy had been uneventful.

There were no contraindications to regional anesthesia, and the parturient consented to epidural labor analgesia using the LORA technique when she had reached about 3 cm of cervical dilatation. Using a TUORen epidural kit, a 16-gauge epidural needle was introduced with a piercing depth of 4 cm and the epidural space was identified with a loss of resistance to 2–3 ml of air technique. The epidural catheter was inserted via a median approach while in lateral decubitus in the L3-4 vertebral interspace and a placement depth of 5 cm. An epidural test dose consisting of 2.5 ml of 2% lidocaine (50 mg) was administered. Epidural analgesia consisted of 10 ml of 0.75% ropivacaine and 50 µg of sufentanil, diluted to 100 ml with saline, and the loading dose was 6 ml. Patient-controlled epidural analgesia (PCEA) was used to maintain analgesia, with 6 ml administered at 30-min intervals. When the analgesia was 15 min, the analgesia reached a satisfactory level of T10, and the whole analgesia procedure lasted for 2 h without any side effects. The epidural block was effective in reducing labor discomfort, and no refills or using emergency pain medications were administered during this period. Then she gave birth to a healthy newborn via lateral episiotomy in the left occipital anterior position due to fetal heart rate of 72–145 beats/min and frequent decelerations. The newborn had Apgar scores of 10 and 10 after 1 and 5 min, respectively. The amount of bleeding at delivery was 150 ml, the placenta-fetal membrane detachment was complete, labor was mostly uneventful, total delivery time was 12 h, routine perineal sutures were used, and 32.5 IU of oxytocin were administered both during and after delivery.

Two hours later, as she prepared to get out of bed, she suddenly felt stiff and sore in her neck and the pain radiated to the top of the head. She described the discomfort in her neck as stuffy and distending, giving the pressure a 7 out of 10. It was the ‘‘worst pain’’ of her life. Her sore neck and headache got worse when she was standing or sitting and went away completely when lying flat. She had trouble walking since her neck was so painfully stiff that she could only hold it in one position. There was no fever and no associated nausea, emesis, photophobia, phonophobia, visual changes, or other neurologic symptoms. Neurologic examination was normal and she had no history of headache.

**Fig. 1:**
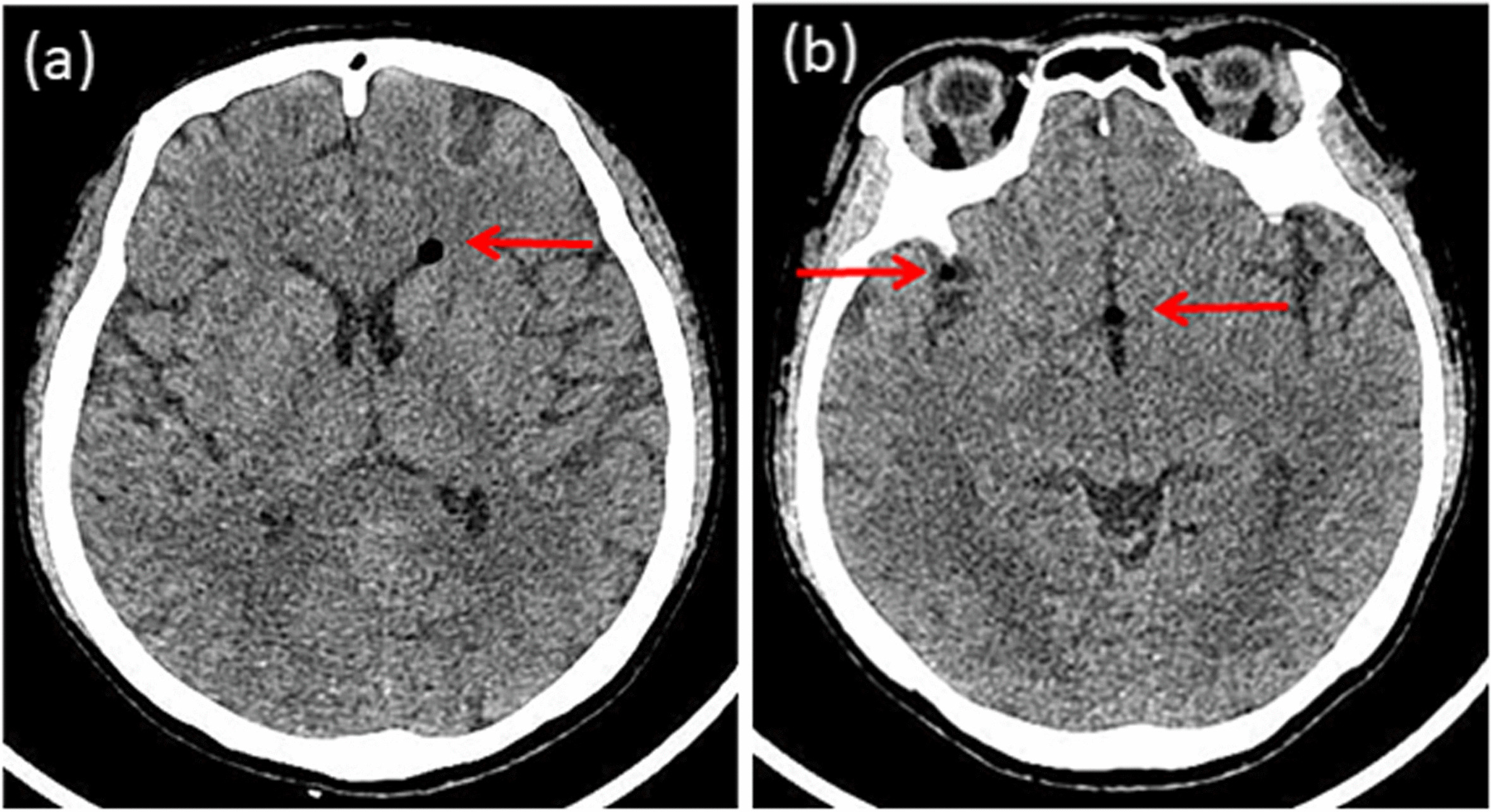
(**a**) and (**b**) Head non-contrast computed tomography images showed scattered gas density shadows in the brain (arrows)

Because the headache presented as an acute attack and was associated with a change in location, we initially considered a low ICP headache and started treatment with 3,000 ml of intravenous fluids daily and oral analgesics as required. The parturient's unremitting headache prompted a neurology consultation. An emergent non-contrast computed tomography (CT) of the head showed scattered gas density shadows in the brain (CT values range from -982Hu to -464Hu) without other abnormalities (Fig. 1(a) and (b)), leading to the diagnosis of pneumocephalus.

**Fig. 2:**
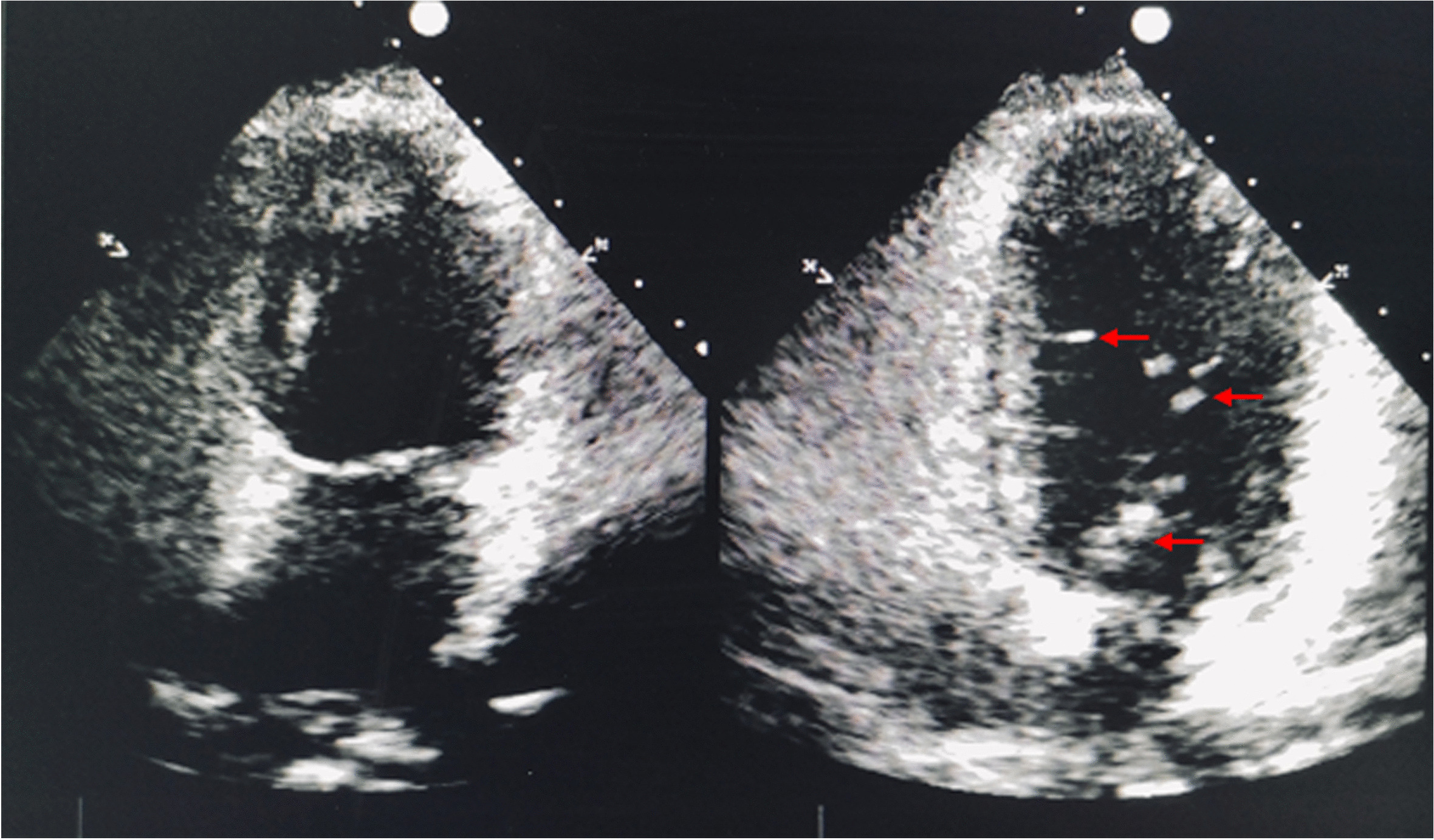
Contrast echocardiography of right ventricle indicated that a small amount of microbubbles (5–6/frame) could be detected in the left heart in the sixth cardiac cycle after Valsalva maneuvers. Microbubbles are noted with arrows

However, we were still unsure whether pneumocephalus might explain the parturient's neck stiffness and position-related headache. Therefore, we did not think PDPH could be completely excluded out. Owing to the consideration of whether the parturient had a possible intracranial air embolism, evidence of a right-to-left shunt was sought. Echocardiography only showed tricuspid few regurgitations. Contrast echocardiography of right ventricle indicated that a small amount of microbubbles (5–6/frame) could be detected in the left heart in the sixth cardiac cycle after Valsalva maneuvers (Fig. 2). In this regard, we believed that microbubbles detected in the left heart after Valsalva maneuvers might have come from the level of the pulmonary arteries, and we were concerned about the possibility of pulmonary embolism in the parturient. It was unfortunate she refused CT-pulmonary angiography (CTPA) because of the risks.

On top of that, we also gave the parturient a non-contrast magnetic resonance imaging (MRI) of the brain. Magnetic resonance plain scan, diffusion weighted imaging (DWI), and magnetic resonance angiography (MRA) of the head without contrast did not reveal any significant abnormalities.

We administered 3,000 ml of intravenous fluids daily, oxygen through mask, and oral analgesics as required while maintaining the parturient in a strict 10-degree head-down position. After 6 days of treatment, she continued to feel a distending pain in her head and a sore neck when she was out of bed, although they were not as bad as they had been. Her headache was fully gone after 2 weeks of lying bed after giving birth. A neurological evaluation performed 4 weeks after discharge was uneventful. She did not have a headache of any kind in the subsequent 6 months since her most recent one.

## Discussion and conclusions

Pneumocephalus, also known as intracerebral aerocele or pneumatocele is defined as the presence of gas within any of the intracranial compartments (intraventricular, intraparenchymal, subarachnoid, subdural, and epidural) of the cranial vault [5], which is usually associated with the disruption of the skull after head and facial trauma and neurosurgery or otorhinolaryngological procedures, and rarely occur in natural labor. This phenomenon was first discovered by Thomas in 1866 during the autopsy of a trauma patient [6]. Then Chiari [7] reported a patient who had pneumocephalus suffering from the complications of chronic septic sinusitis. The term pneumocephalus was coined and first used by Wolff in 1914 [8].

There are two theories for the development of pneumocephalus, “ball valve” theory and “inverted bottle” effect. The “ball valve” theory suggests that positive pressure events like coughing, sneezing, and Valsalva maneuvers force air through a cranial defect, which subsequently prevents the air from escaping on its own. Significant resistance to the outflow of air leads to tension pneumocephalus. In the “inverted bottle” effect, drainage of cerebrospinal fluid (CSF) leads to a negative ICP gradient which is relieved by the influx of air [5].

Pneumocephalus may be asymptomatic or present with signs of increased intracranial pressure. Headache and altered consciousness are the most frequent symptoms of pneumocephalus [9]. Subarachnoid air can cause significant irritation; so as little as 2 mL of subarachnoid air has been reported to cause headache [10].

Clinically, it was apparent that the parturient's condition was not simply intracranial hypertension, thus, her symptoms did not support the potential diagnosis of tension pneumocephalus. Her neck stiffness and headache symptoms were consistent with low ICP syndrome; the pneumocephalus could not account for them. Then we ruled out neck pain caused by muscle spasm since there were no abnormalities in the skin or temperature of the neck, no negative pressure discomfort in the paravertebral muscles, no striated alterations on muscle palpation, and no radiating pain in the upper extremities. Next, we questioned the parturient about any changes in addition to the neck stiffness. Reviewing this with the parturient, she told us that the neck stiffness was accompanied by persistent tinnitus in one of her ears for the first 5 days (the specific side had been forgotten). She did not pay attention to the severity because it did not disrupt her daily life and found that it worsened when she was not well rested. Long regarded as a marker of PDPH, the headache should be positional (i.e., much better lying than upright), and its location can be frontal, occipital, or posterior at the back of the neck. It frequently comes with nausea, neck stiffness, tinnitus, or dizziness [11]. A dural puncture allows CSF to leak out of the central neuraxial space, leading to a decrease in intracranial CSF volume and low ICP. The postural nature of the headache and cranial nerve symptoms is caused by the significantly reduced CSF volume in the intrathecal sac, which permits a relative “sagging” in the upright posture and tension on the cranial nerves and blood vessels [12]. The link between the endolymph and the CSF may cause muted hearing, and pressure or tension on the CN VIII (eighth cranial nerve) may cause ears to “buzz” [13]. This information led us to prefer the diagnosis of PDPH combined with pneumocephalus.

This parturient received epidural anesthesia using the LORA technique. Epidural analgesia is a widely used therapy for labor analgesia around the world. The definitive point for determining the epidural space is either LORA or saline (LORS). Confirmation of entry into the epidural space, as the name implies, occurs upon recognition of the sudden release of resistance, usually of air as the advancing needle emerges from the ligamentum flavum [14]. Headache after epidural anesthesia is often attributed to low ICP, due to leakage of CSF [15]. Parturients have approximately a 1.5% risk of accidental dural puncture with epidural insertion. Of these, around half will result in PDPH [16], and although there is no significant difference in the incidence of dural puncture, the incidence of headache is much higher (more than six times) in patients using the LORA approach than the LORS technique [17]. In view of this, the formation of pneumocephalus in this parturient could be explained by an unintentional dural puncture (UDP) that resulted in CSF leakage and the resultant low ICP headache, which was restored by the introduction of air.

**Fig. 3:**
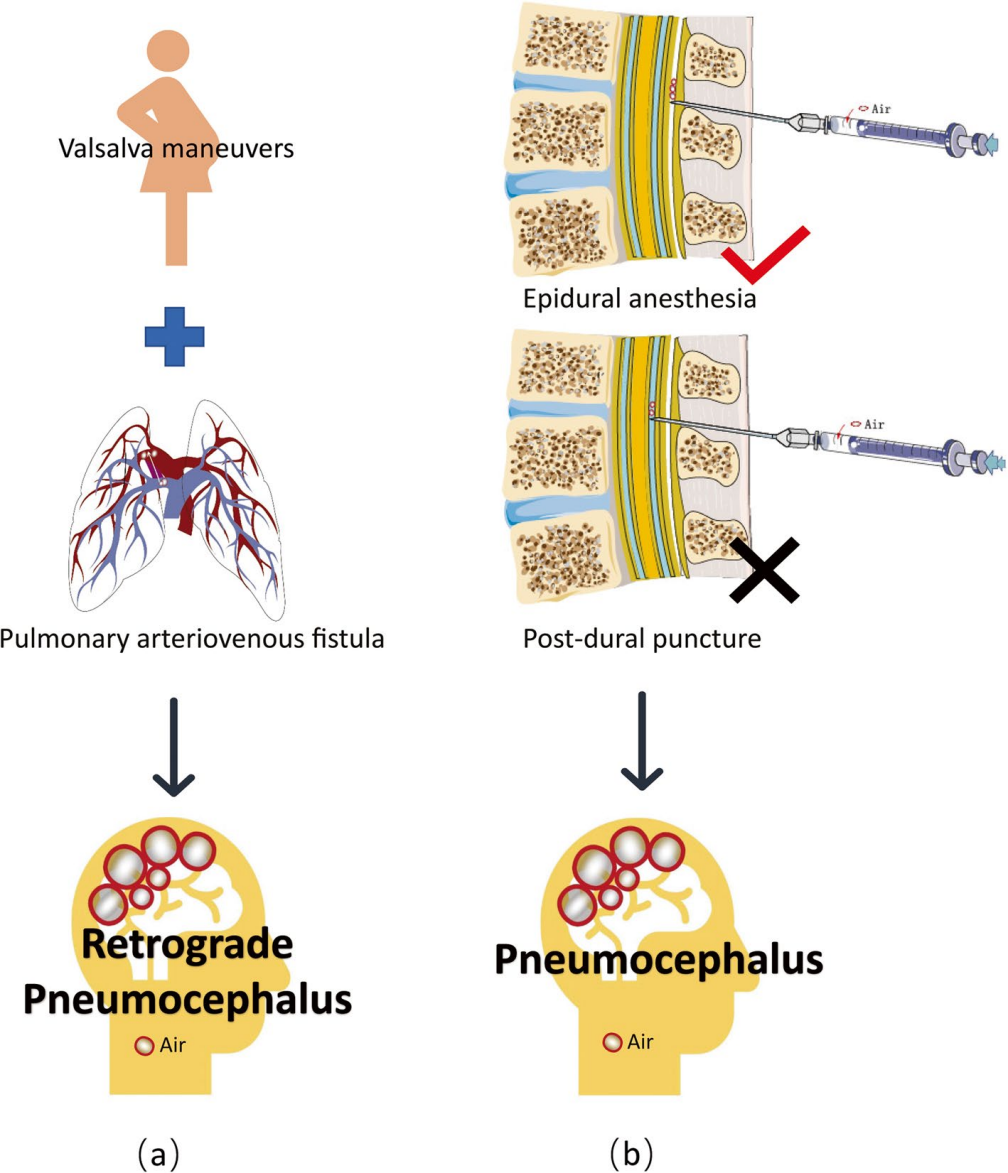
(**a**) The formation of intracranial air embolism and retrograde pneumocephalus. (**b**) Pneumocephalus-related headache immediately onsets following the dural puncture and injection of air when epidural anesthesia using the loss of resistance to air technique

In this parturient, other headache-causing etiologies were also taken into account. Pneumocephalus morbidity has been linked to attempted epidural anesthesia utilizing the LORA approach. The immediate onset of headache following the dural puncture, injection of air and the subsequent presence of intracranial air confirm the diagnosis of pneumocephalus-induced headache (Fig. 3(b)). Nevertheless, the headache in this case was found 10 h after the epidural, not immediately after attempted epidural anesthesia using the LORA technique.

Retrograde pneumocephalus occurs as a result of the air entering the right atrium to the brain. Because natural childbirth involves Valsalva maneuvers and this parturient may have a pulmonary arteriovenous fistula, cerebral air embolism can be discovered if these two criteria are met, and the uterine venous sinuses also provide optimum conditions for air to enter the veins (Fig. 3(a)). Also, she has a RoPE (Risk of Paradoxical Embolism) score of 9, which indicates a risk of paradoxical embolism. However, we were unable to determine this due to lack of CTPA. Furthermore, we believe that the ensuing retrograde pneumocephalus causes intracranial hypertension rather than low ICP.

Despite conventional belief that PDPH may be a severe, debilitating short-term headache, recent research shows an increased risk of mid- and long-term effects, including chronic back pain and headaches, decreased breastfeeding rates, postpartum depression, and post-traumatic stress disorder, 1 year after giving birth [18]. Alarmingly, Epidural puncture or ‘nicking’ of the dura may not have been noticed in > 30% of post-epidural PDPHs [19]. Therefore, post-neuraxial follow-up should be given to all patients regardless of their symptoms [18], especially if they have a known epidural UDP. Parturients with mild PDPH symptoms within the first 24–48 h can be managed conservatively (e.g., recumbent position, appropriate hydration, non-opioid analgesics, and opioids for severe breakthrough pain) for symptomatic relief whereas parturients exhibiting severe PDPH symptoms should receive definitive epidural blood patch treatment, especially when affecting the mother–child interaction [12].

In those parturients whose headaches are not getting better or progressively getting worse, further neurological workup or neurological consultation including brain imaging is advised. Head plain CT is the gold standard investigation in the diagnosis of pneumocephalus. It can detect even 0.55 ml of intracranial air, whereas a skull radiograph requires at least 2 ml [20]. MRI may also be useful, but not as sensitive as a CT scan in the diagnosis of pneumocephalus. Moreover, air may be mistaken for flow voids or blood products, and it appears dark in almost all MR sequences [21].

The danger of pneumocephalus should not be lower estimated. The presence of air can cause seizures by irritating the cerebral cortex [21]. The different distribution of intracranial air bubbles causes different symptoms of neurological deficits [4]. Thus early prevention, recognition and diagnosis of pneumocephalus are essential.

As soon as pneumocephalus is confirmed, the primary treatment goal is to keep air from entering the body, which includes placing the parturient in 30 degrees Fowler position, avoiding Valsalva maneuvers, high flow oxygen therapy via a face tent or 100% non-re-breather mask with absolute avoidance of positive pressure [22] and hyperbaric oxygen therapy [21].

Last but not least, it is critical to emphasize that postpartum headaches should never be solely attributed to anesthetic and should always be thoroughly investigated for obstetric reasons and comorbidities. Positional headaches after epidural anesthesia may not indicate low ICP alone; it may combine with pneumocephalus, particularly when using the LORA technique. Obstetricians and anesthesiologists should be made aware of this phenomenon. If headaches are accompanied by seizures or focal neurological impairments, head CT is essential to discover other conditions like pneumocephalus.

## Data Availability

The datasets analyzed during the current study are not publicly available due to protection of the patient’s privacy but are available from the corresponding author on reasonable request (email: lguogang@163.com).

## Abbreviations

ICP: Intracranial pressure
LORA: Loss of resistance to air
PDPH: Post-dural puncture headache
PCEA: Patient-controlled epidural analgesia
CT: Computed tomography
CTPA: Computer tomography pulmonary angiography
MRI: Magnetic resonance imaging
DWI: Diffusion weighted imaging
MRA: Magnetic resonance angiography
CSF: Cerebrospinal fluid
LORS: Loss of resistance to saline
UDP: Unintentional dural puncture
RoPE: Risk of paradoxical embolism
